# An alternative physiological role for the EmhABC efflux pump in *Pseudomonas fluorescens *cLP6a

**DOI:** 10.1186/1471-2180-11-252

**Published:** 2011-11-15

**Authors:** Abigail A Adebusuyi, Julia M Foght

**Affiliations:** 1 Department of Biological Sciences, University of Alberta, Edmonton, Alberta, T6G 2E9, Canada

## Abstract

**Background:**

Efflux pumps belonging to the resistance-nodulation-division (RND) superfamily in bacteria are involved in antibiotic resistance and solvent tolerance but have an unknown physiological role. EmhABC, a RND-type efflux pump in *Pseudomonas fluorescens *strain cLP6a, extrudes hydrophobic antibiotics, dyes and polycyclic aromatic hydrocarbons including phenanthrene. The effects of physico-chemical factors such as temperature or antibiotics on the activity and expression of EmhABC were determined in order to deduce its physiological role(s) in strain cLP6a in comparison to the *emhB *disruptant strain, cLP6a-1.

**Results:**

Efflux assays conducted with ^14^C-phenanthrene showed that EmhABC activity is affected by incubation temperature. Increased phenanthrene efflux was measured in cLP6a cells grown at 10°C and decreased efflux was observed at 35°C compared with cells grown at the optimum temperature of 28°C. Membrane fatty acids in cLP6a cells were substantially altered by changes in growth temperature and in the presence of tetracycline. Changed membrane fatty acids and increased membrane permeability were associated with ~30-fold increased expression *of emhABC *in cLP6a cells grown at 35°C, and with increased extracellular free fatty acids. Growth *of P. fluorescens *cLP6a at supra-optimal temperature was enhanced by the presence of EmhABC compared to strain cLP6a-1.

**Conclusions:**

Combined, these observations suggest that the EmhABC efflux pump may be involved in the management of membrane stress effects such as those due to unfavourable incubation temperatures. Efflux of fatty acids replaced as a result of membrane damage or phospholipid turnover may be the primary physiological role of the EmhABC efflux pump in *P. fluorescens *cLP6a.

## Background

Efflux pumps of the resistance-nodulation-division (RND) superfamily contribute to antibiotic resistance, virulence and solvent tolerance in Gram-negative bacteria [1-3]. The clinical significance of RND efflux pumps and their relevance to bioremediation necessitate understanding the factors influencing their expression and activity. Previous studies seeking the inducers of genes encoding RND efflux pumps focussed on known substrates of the pumps [4,5]. However, such studies showed that substrates are often not inducers, and the pumps are present in bacterial cells that have not been exposed to antibiotics or solvents [5-7]. Furthermore, genes encoding RND efflux pumps can be induced by stress responses such as ribosome disruption or membrane-damaging agents [4,7-9]. These observations suggest a physiological function for RND efflux systems beyond the transport of antibiotics or solvents. Knowledge of the primary physiological role for such pumps in Gram-negative bacteria may aid development of new methods to combat antibiotic resistance [7] and improvement of biocatalytic processes such as production of enantio-pure compounds from hydrocarbons or bioremediation of polycyclic aromatic hydrocarbon (PAH) pollutants.

The first step in identifying the primary physiological role of RND efflux pumps is to determine the effect of physico-chemical factors on efflux pump activity and expression of genes that encode them. A common physical factor affecting the composition and physiology of bacterial cells is incubation temperature [10,11], which influences bacterial cell membrane fatty acid (FA) composition [11,12]. Altered membrane FA composition is an adaptation mechanism used by bacteria to compensate for changes in membrane fluidity caused by physiological or biochemical stress. Fluidity of the membrane affects the interaction of lipids and proteins (including RND efflux pumps) anchored in the membrane and in turn permeation and transport of hydrophobic molecules across the membrane [11,13,14]. Changes in the resistance of cells grown at different temperatures to various environmental stresses have been reported [10]. However, increased resistance to antibiotics, environmental stresses or membrane-damaging agents has not previously been linked to the effect of growth temperature on increased activity of efflux pumps or expression of their genes.

*Pseudomonas fluorescens *LP6a, an isolate from petroleum condensate-contaminated soil, utilizes PAHs such as naphthalene, phenanthrene and anthracene as sole carbon source [15,16]. A RND-type efflux pump (EmhABC) in this strain that extrudes hydrophobic antibiotics and PAHs has been described previously [17-19], where EmhA is the membrane fusion protein, EmhB is the RND protein and EmhC is the outer membrane protein [18]. The EmhABC efflux pump in *P. fluorescens *is a good model for investigating a physiological role for RND-type efflux pumps because it extrudes PAHs considered non-toxic to the cells as well as hydrophobic antibiotics [17] and its expression is not induced by its PAH substrates [18]. PAH transport can be monitored in the absence of PAH metabolism [18] by using strain cLP6a, a cured strain of *P. fluorescens *LP6a lacking the PAH catabolic plasmid pLP6a [16]. Comparing the properties of cLP6a with its *emhB *disruption mutant strain cLP6a-1 [18] allows inference of a physiological role for the RND efflux pump EmhABC based on the effect of growth temperature, antibiotics or PAHs on its activity and expression in relation to membrane FA changes.

## Methods

### Bacterial strains and growth conditions

*P. fluorescens *cLP6a is a cured strain of the wild type *P. fluorescens *strain LP6a that lacks the catabolic plasmid pLP6a [16] and cannot metabolize PAHs. Strain cLP6a-1 is an *emhB *disruption mutant of the cured strain [18]. Strains were grown to stationary phase (unless otherwise indicated) in 100 ml of trypticase soy broth (TSB) (Difco) with gyratory shaking at 200 rpm at 10°C, 28°C (the optimal growth temperature; [15]) or 35°C. TSB inoculated with strain cLP6a-1 contained kanamycin (Sigma) at 25 μg ml-^1 ^to maintain the gene disruption. Growth was measured as optical density at 600 nm (OD_600_) using an Ultrospec 3100 pro UV/Visible spectrophotometer (GE Healthcare Bio-Sciences), diluting the TSB blank and culture sample with distilled water as necessary. Naphthalene and phenanthrene were added at a final concentration of 5 mmol l^-1^, either dissolved in N,N-dimethylformamide (ACS grade, Anachemia) and added to cultures used for RNA extraction or added as a suspension of crystals to cultures used for fatty acid extraction.

### Phenanthrene efflux assay

Efflux of [9-^14^C]phenanthrene (96.5% radiochemical purity; Amersham) was determined using a rapid centrifugation method [17] conducted at room temperature (~22°C). The final concentration of radiolabeled plus unlabeled phenanthrene in the assay medium was 6.4 μM, which corresponds to 90% of its aqueous solubility limit at that temperature and ensures that insoluble phenanthrene does not confound measurement of cell-associated radiolabel. *P. fluorescens *cLP6a and cLP6a-1 cells were harvested by centrifugation, washed once with potassium phosphate buffer [pH 7] and re-suspended in the same buffer at room temperature at an OD_600 _of 1.0. Cell suspensions were used immediately in the rapid assay to prevent long-term FA composition changes, and phenanthrene efflux was measured over a period of only 25 min. At time zero radiolabeled phenanthrene was added to the cell suspension and thereafter samples were withdrawn at timed intervals, collecting the cells by using a microfuge. The concentration of phenanthrene in the cell pellet (μmol/g) was calculated from the amount of ^14^C in the pellet fraction, the initial phenanthrene concentration and the cell dry weight as previously described by Bugg et al. [17]. Sodium azide (Fisher Scientific) was added 9 min into the assay to a final concentration of 120 mM as an inhibitor of active transport [17]. All efflux assays were performed using independent triplicate cultures. Steady state concentrations pre- and post-azide addition were calculated and statistically evaluated by analysis of variance (ANOVA) in Excel.

### Antibiotic sensitivity assays

The minimum inhibitory concentration (MIC), the lowest concentration of antibiotic that inhibits growth, was measured as turbidity (OD_600_) using a Powerwave XS spectrophotometer (BioTek). The MICs of tetracycline, streptomycin, nalidixic acid, erythromycin and chloramphenicol were determined using the microtiter broth dilution method [20] for *P. fluorescens *cLP6a and cLP6a-1 grown at 10°C, 28°C or 35°C.

### RNA extraction

*P. fluorescens *cLP6a cells were grown in TSB to logarithmic, stationary or death phase at 28°C; to stationary phase at 10°C, 28°C or 35°C; or to stationary phase in the presence of antibiotics (chloramphenicol or tetracycline at ¼ MIC) or PAHs (naphthalene or phenanthrene at 5 mmol l^-1^). At point of harvest, 10 ml of culture was stopped by adding 1.25 ml of ice-cold ethanol/phenol solution (5% water-saturated phenol, in ethanol). Total RNA was immediately extracted from the harvested cultures using MasterPure™ RNA Purification Kit (Epicentre Biotechnologies) according to the manufacturer's instructions. Total RNA recovered was dissolved in 100 μl of nuclease-free water and treated with 10 μl of 10× DNase I buffer and 10 units of RNase-free DNase I (Ambion): the reaction was incubated at 37°C for 30 min, stopped with 5 μl of 50 mM EDTA [pH 8] and then 1 μl of SUPERase•In (Ambion) was added before storage at -80°C. The purity and concentration of the RNA extracted from each culture sample was determined using an Agilent 2100 bioanalyzer (Agilent Technologies).

### Reverse-transcription-PCR (RT-PCR)

A RNA-primer hybridization mix containing 2 μl DNase-treated total RNA and 10 ng/μl random hexamer primers (Invitrogen) was incubated in a thermocycler at 70°C for 10 min followed by 25°C for 10 min. The 60 μl cDNA synthesis mixture contained the RNA-primer mix, 0.5 mM dNTP mix, 1 × first strand buffer (Invitrogen), 10 mM dithiothreitol, 0.5 U/μl SUPERase•In (Ambion) and 6.7 U/μl SuperScript III reverse transcriptase (Invitrogen). The mixture was incubated at 25°C for 10 min, 37°C for 60 min, 42°C for 60 min and then at 70°C for 10 min to inactivate the SuperScript III. cDNA was stored at -80°C until used for real-time PCR.

### Primer design for quantitative real-time PCR (qPCR)

Primers were designed for qPCR using Primer Express^® ^Software v3.0, which considers factors such as amplicon size, homology with other genes, secondary structure and the estimated duplex melting temperature (*T*_*m*_). Primers were designed using partial sequences retrieved from GenBank (http://www.ncbi.nlm.nih.gov/genbank/) for *emhA *(AAQ92180), *emhB *(AAQ92181) and *emhC *(AAQ92182) of *P. fluorescens *cLP6a [18] and the 16S rRNA gene *of P. fluorescens *pf0-1 (NC_007492) [21], the latter being used as the endogenous control. Primer pairs designed for each gene are listed in Table 1.

**Table 1:** Primers for qPCR analysis

Gene	Forward primer (5' → 3')	Reverse primer (5'→ 3')
***emhA***	CGGTGAGCCGTCAGGAATAC	TTGATCTGGGCGCTTTGC
***emhB***	GTCCCACTGGCGATTTCC	CCGTGATCATACCGCCAATAA
***emhC***	GATCGCCTGGCGCAACT	CTTTCGCAGTCTGCTCATTCC
**16S rRNA**	GGAGACTGCCGGTGACAAACT	TGTAGCCCAGGCCGTAAGG

### RT-qPCR

qPCR of cDNA was performed using an ABI 7500 Fast Real-Time PCR System (Applied Biosystems). Each 10-μ1 RT-qPCR reaction mixture containing 2.5 μl cDNA and 0.4 μM of each corresponding primer specific for target genes or the endogenous control was incubated with a reaction mixture (Molecular Biology Services Unit, Edmonton, Canada) comprising 5 μl 2 × qPCR reaction mix with SYBR Green (Molecular Probes) as the detection dye and ROX (Invitrogen) as a normalizing dye. The PCR conditions consisted of a denaturation cycle at 95°C for 2 min, followed by 40 cycles at 95°C for 30 s and 60°C for 1 min, and a dissociation cycle at 95°C for 15 s, 60°C for 1 min, 95°C for 15 s and then 60°C for 15 s. The melting curve generated at the end of real-time PCR cycles was analysed to confirm the absence of nonspecific double stranded DNA-SYBR Green hybrids.

RT-qPCR data analysis was performed using the gene expression study function of the ABI 7500 software v2.0 (Applied Biosystems). The fluorescence of SYBR Green is measured against ROX at the end of each PCR cycle in the ABI 7500 Fast Real-Time PCR System. The comparative C_T _method (2^-ΔΔCT^) was used to calculate the relative quantities of nucleic acid sequence of target genes in each sample [22]. C_T _(threshold cycle) is the fractional cycle number at which the SYBR Green fluorescence passes the baseline signal [22]. The expression levels of target genes were normalized against that of the 16S rRNA gene (endogenous control). RNA obtained from *P. fluorescens *cLP6a cultures grown at 28°C to stationary phase was used as the calibrator sample in this study. Statistical analysis of data was performed using ANOVA (Excel 2007).

### Membrane integrity assay

Membrane integrity of *P. fluorescens *cLP6a cells grown to stationary phase at 10°C, 28°C or 35°C was determined using a modification of the method described by Niven and Mulholland [23]. Cell samples (1 ml) were harvested by centrifugation, re-suspended in 1 ml of phosphate-buffered saline and adjusted to an OD_600 _of 1.0. Propidium iodide (PI; Invitrogen), either alone or with the membrane-disrupting agent cetyltrimethylammonium bromide (CTAB; Sigma), were added to final concentrations of 30 μmol l^-1 ^and 1 μmol l^-1 ^respectively; untreated cells were included as parallel controls. After 30 min incubation at room temperature, fluorescence of 100-μl cell samples was measured in a 96-well microplate using a Synergy HT Multi-mode Microplate Reader (BioTek) at excitation and emission wavelengths of 500 nm and 600 nm respectively.

### Phospholipid fatty acid (FA) extraction and identification

Total cell lipids were extracted using the Bligh-Dyer method [24] modified by White and Ringelberg [25] from 10 mg lyophilized cLP6a or cLP6a-1 cells grown to stationary phase at different temperatures or in the presence of antibiotics (at 1/4 MIC) or PAHs (5 mmol l^-1^). Fatty acid methyl esters (FAME) were prepared from extracted total lipids using mild alkaline methanolysis [26], dried under a stream of N_2 _and re-dissolved in 500 μl chloroform (HPLC grade, Fisher Scientific). FAME were analysed by gas chromatography with mass spectrometry (GC-MS) on an Agilent 6890N GC with a model 5973 inert mass selective detector (Agilent) fitted with an Agilent HP-5MS capillary column (30 m × 0.25 mm ID, 0.25 μm film thickness; J + W Scientific). Helium was used as the carrier gas with a temperature program of 150°C (1 min) increasing to 190°C at 1.5°C min^-1^, then 25°C min^-1 ^to 290°C (held for 4 min). Sample peaks were compared to Bacterial Acid Methyl Ester Mix standards (Supelco, Sigma Aldrich) and quantified by calculating individual FAME peak areas as a percentage of the total FAME in each sample [27].

### Free FA assay

*P. fluorescens *strains cLP6a and cLP6a-1 cultures grown to stationary phase at 10°C, 28°C or 35°C were harvested by centrifugation. The culture supernatants were filtered using a 0.22 μm Millex-GS filter unit (Millipore), then 50 μl of the filtrate was assayed for free FA using a free fatty acid quantification kit (Abcam) according to the manufacturer's protocol.

## Results

### EmhABC enhances growth at supra-optimal temperature

**Figure 1:**
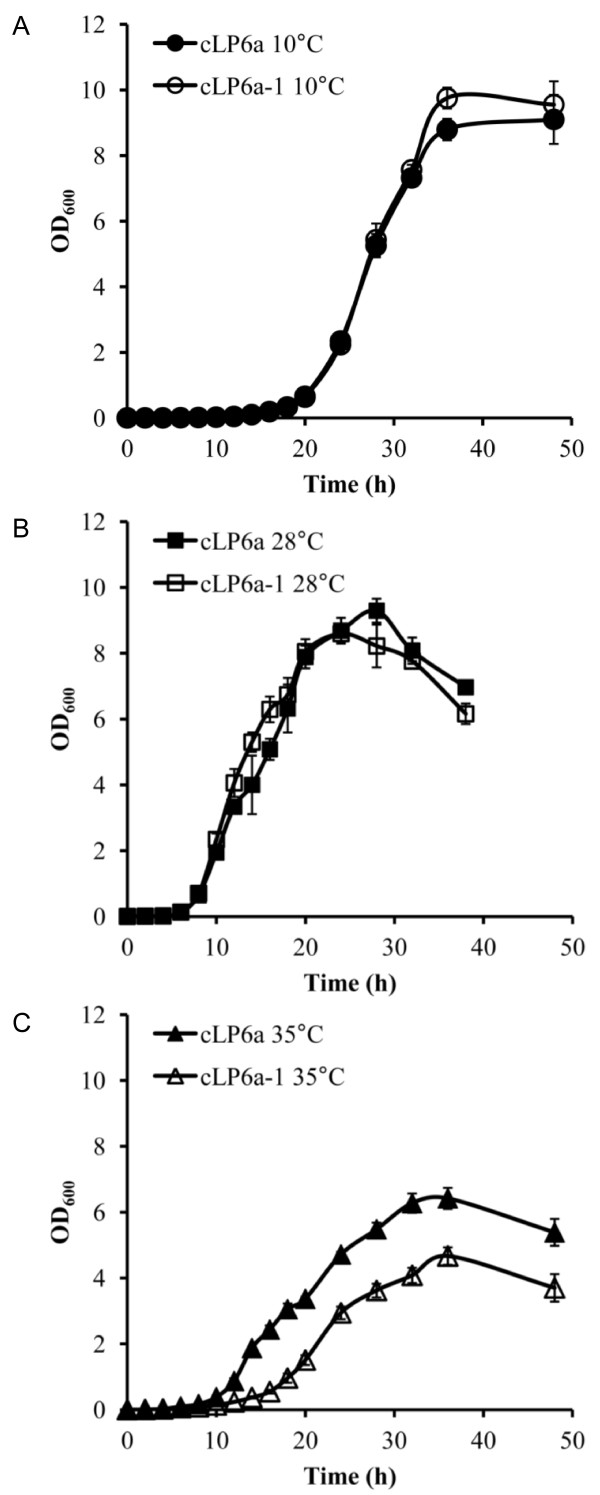
**Growth curves of *P. fluorescens *strains cLP6a and cLP6a-1**. Growth of *P. fluorescens *strains cLP6a and cLP6a-1 at (a) 10°C, (b) 28°C or (c) 35°C determined as OD600 Each data point is the mean of three independent cultures, and error bars, where visible, indicate the standard deviation.

Growth curves for *P. fluorescens *strains were determined at 10°C, 28°C or 35°C to allow sampling at the appropriate phase of growth in subsequent studies. The optimum growth temperature for wild type *P. fluorescens *LP6a is 28°C [15], 10°C is a growth-permissive sub-optimal temperature, and 35°C is ~2°C below the maximum growth temperature of *P. fluorescens *LP6a wild type. Strains cLP6a and cLP6a-1 grown in seed cultures at 28°C were transferred to fresh medium and incubated at 10°C, 28°C or 35°C and growth was monitored for 48 h. The growth curves of cLP6a and cLP6a-1, measured as OD_600_, were similar to each other at 10°C (Figure 1a) and at 28°C (Figure 1b). The lag phases of both cLP6a and cLP6a-1 were longer at 10°C than at 28°C but the maximum OD_600 _achieved was greater at 10°C. The maximum OD_600 _achieved by cLP6a and cLP6a-1 was lower at 35°C and growth of the two strains was dissimilar (Figure 1c). The growth yield for strain cLP6a-1 at 35°C was about half that measured at 10°C and 28°C, and ~70% that of strain cLP6a at 35°C. Thus, disruption *of emhABC *in strain cLP6a-1 impaired its growth rate and cell yield at the supra-optimal temperature.

### Phenanthrene efflux by EmhABC is affected by incubation temperature

To measure activity of the EmhABC efflux pump, a rapid efflux assay [17] was performed using ^14^C-phenanthrene. In the efflux assay, suspensions of cLP6a and cLP6a-1 harvested at stationary phase were incubated with ^14^C-phenanthrene at a concentration below its aqueous solubility limit, to avoid any effects of dissolution on phenanthrene bioavailability. Partitioning of phenanthrene into the cells is very rapid, achieving steady state in less than 1 min [17]. At timed intervals, the radiolabel associated with the cell pellet is measured, and the steady state concentration is the sum of efflux and partitioning of phenanthrene. A significant increase in the concentration of phenanthrene associated with the cell pellet after addition of sodium azide indicates inhibition of active efflux, resulting in phenanthrene accumulation in the cell. A constant high concentration of phenanthrene in the pellet both before and after azide addition indicates absence of efflux. Therefore, the relative activity of EmhABC at different incubation temperatures can be determined by comparing the difference between the steady state cell-associated phenanthrene concentrations during active efflux (pre-azide addition for strain cLP6a) and in the absence of efflux (in strain cLP6a-1 and post-azide addition for strain cLP6a); the greater the difference between phenanthrene concentrations in the pellets, the greater the efflux pump activity and consequently the more phenanthrene extruded from the cells. Importantly, because the centrifugation assay is so rapid (~25 min duration), the observed effects must be due to existing efflux pumps and membrane fatty acid (FA) composition rather than being influenced by induction *of emhABC *transcription or long-term membrane modifications through *de novo *synthesis of FA.

**Figure 2:**
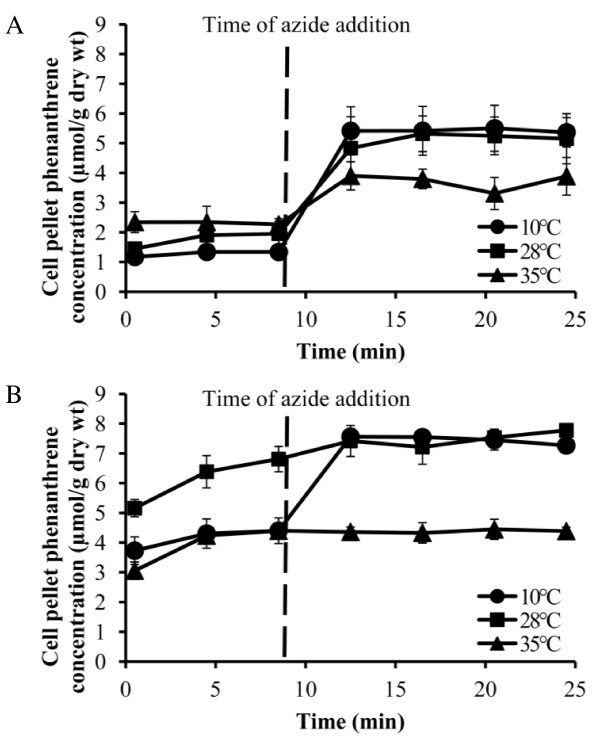
**Phenanthrene partitioning into *P. fluorescens *strains cLP6a and cLP6a-1**. Partitioning of phenanthrene into the cell pellet of *P. fluorescens *strains, determined using a rapid efflux assay: (a) strain cLP6a grown at 10°C, 28°C or 35°C; (b) strain cLP6a-1 grown at 10°C, 28°C or 35°C. The vertical dashed line indicates the addition of azide (120 mM). Each data point is the mean of three independent experiments, and error bars, where visible, indicate the standard deviation.

Because incubation temperature affects FA composition and fluidity of membranes, which in turn can affect protein-lipid interactions and integral membrane protein activity [11], we determined the effect of growth temperature over a 25°C range on subsequent phenanthrene efflux activity. The cell-associated phenanthrene prior to azide addition was 1.34 ± 0.19 μmol/g, 1.93 ± 0.34 μmol/g and 2.30 ± 0.36 μmol/g in cLP6a cells grown at 10°C, 28°C and 35°C respectively, indicating reduced efflux activity with increasing growth temperature. Consistent with previous work [18], cLP6a cells grown at 28°C exhibited active efflux of phenanthrene (Figure 2a): the steady state concentrations of phenanthrene associated with the cell pellet before (1.93 ± 0.34 μmol/g ) and after (5.28 ± 0.56 μmol/g ) azide addition were significantly different (*P *< 0.0001).

Efflux assays were also performed with the *emhB *disruption strain cLP6a-1 (Figure 2b) to determine the steady state concentration of phenanthrene in the absence of efflux in the cells. As expected, there was no evidence of phenanthrene efflux by mutant cLP6a-1 at 28°C and 35°C, as the steady state concentrations of cell-associated phenanthrene were unchanged before and after azide addition. Notably, the cell-associated phenanthrene prior to azide addition was significantly greater in cLP6a-1 cells grown at 28°C (6.60 ± 0.50 μmol/g) than in the parallel cLP6a cells (1.93 ± 0.34 μmol/g; *P *< 0.0001) (Figure 2). Thus, EmhABC is the sole efflux system responsible for phenanthrene efflux in cLP6a cells grown at 28°C and 35°C. The cell-associated phenanthrene concentration in cLP6a-1 cells grown at 35°C before azide addition (4.32 ± 0.19 μmol/g) was significantly lower (*P *< 0.0001) than in cells grown at 28°C (6.60 ± 0.50 μmol/g; Figure 2b), suggesting that phenanthrene partitioning into the cells was affected by changes in membrane FA composition induced by the incubation temperature. Unexpectedly, in cLP6a-1 cells grown at 10°C (Figure 2b) the cell-associated phenanthrene concentrations pre- (4.35 ± 0.42 μmol/g) and post- (7.50 ± 0.16 μmol/g) azide addition were significantly different (*P *< 0.0001), consistent with efflux subsequently inhibited by azide. This observation suggests the activity of another phenanthrene efflux pump(s) present and active at 10°C but not at 28°C. A second efflux pump expressed or active at low temperature would also explain why cLP6a cells grown at 10°C accumulated the lowest measured concentration of cell-associated phenanthrene prior to azide addition (Figure 2a): this could result from the combined activity of EmhB plus the postulated alternate efflux pump at the low temperature.

The difference in cell phenanthrene concentration in the presence and absence of efflux in cLP6a grown at 10°C (6.18 ± 0.002 μmol/g) was significantly greater (*P *< 0.002) than in cLP6a cells grown at 28°C (5.46 ± 0.03 μmol/g). Because a putative pump was likely induced at 10°C in addition to EmhB (Figure 2b), the actual difference in cell pellet phenanthrene concentration due to the activity of EmhB in strain cLP6a grown at this temperature (3.01 ± 0.07 μmol/g) was significantly lower *(P *< 0.001) than in cells grown at 28°C. Similarly the difference in phenanthrene concentrations for strain cLP6a grown at 35°C (2.07 ± 0.06 μmol/g) was less than in cells grown at 28°C. These results indicate that the activity of EmhB was reduced due to sub- or supra optimal incubation temperature. Therefore incubation temperature affects phenanthrene efflux by the EmhB efflux pump.

### Incubation temperature affects sensitivity to antibiotics

The effect of incubation temperature on antibiotic efflux by EmhABC was investigated to confirm the phenanthrene efflux assays. The sensitivity of cLP6a and cLP6a-1 cells grown at 10°C, 28°C or 35°C to various antibiotics was measured indirectly as MICs to test the effect of temperature on efflux of known antibiotic substrates of the EmhABC pump [18,19]. As expected, the *emhB *mutant strain (cLP6a-1) was more sensitive to such antibiotics than strain cLP6a grown at a comparable incubation temperature (Table 2), exhibiting a ≥ 16-fold difference in MIC for chloramphenicol, nalidixic acid and tetracycline, and a 4- to 8-fold difference for erythromycin. Both strains showed similar sensitivity to ampicillin, which is not a substrate of EmhABC [18,19]. Smaller differences in MIC values (<8-fold, or no difference) were observed within a single strain incubated at different temperatures for some antibiotics.

**Table 2:** Antibiotic sensitivity of *P. fluorescens *strains cLP6a and cLP6a-1 incubated at different temperatures

		MIC (μg ml^-1^) *				
		
*P. fluorescens *strain	Growth temperature	AMP	CHL	ERY	NAL	TET
cLP6a	10°C	512	64	128	32	2
	28°C	512	32	128	32	2
	35°C	256	8	64	32	1

cLP6a-1	10°C	512	4	32	2	0.125
	28°C	512	1	8	<1	0.125
	35°C	512	<0.5	8	<1	<0.063

*, AMP, ampicillin; CHL, chloramphenicol; ERY, erythromycin; NAL, nalidixic acid; TET, tetracycline

Antibiotic sensitivity was measured as minimum inhibitory concentration (MIC). Values shown are representative data from two independent experiments.

### *emhABC *expression is affected by incubation temperature and growth phase

**Figure 3:**
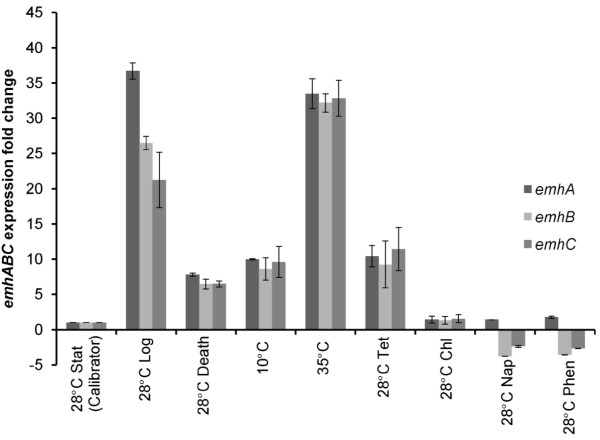
**Expression of *emhABC *efflux genes**. Expression of *emhABC *in *P. fluorescens *strain cLP6a grown to stationary (Stat), logarithmic (Log) or Death phase at 28°C; grown to stationary phase at 10°C or 35°C; grown to stationary phase at 28°C in the presence of chloramphenicol (Chl) or tetracycline (Tet) at 1/4 MIC; or grown to stationary phase at 28°C in the presence of naphthalene (Nap) or phenanthrene (Phen) at 5 mmol l^-1^, determined using RT-qPCR. The values shown are the fold-difference in expression of *emhABC *compared to expression levels in cells grown to stationary phase at 28°C (calibrator = 1). Each bar represents the mean of two independent experiments performed in duplicate. Error bars, where visible, indicate the average deviation.

Changes in the activity of EmhABC in cLP6a cells grown at different temperatures could reflect differential expression *of emhABC*, differential EmhABC translation or changes in the membrane physiology of the cells as a result of deviation from the normal growth temperature. Thus we determined the effect of incubation temperature on the expression *of emhABC *and on the cell membrane physiology. It is assumed that the *emhABC *genes form an operon based on their homology to the *ttgABC *and *mexAB-OprM *efflux operons [18]. Expression of the *emhABC *genes in cLP6a cells incubated at different temperatures and grown to different phases was determined using RT-qPCR to identify the condition(s) that induce *emhABC *transcription. The reference level of expression (i.e., calibrator) was defined as that exhibited by cLP6a cells grown to stationary phase at 28°C. Expression at 28°C was dependent on growth phase: *emhABC *genes were induced ~20-35 fold in log phase cells, and ~6-fold in death phase cells (Figure 3). Sub- and supra-optimal incubation temperature also increased expression ~10-fold at 10°C and ~32-fold at 35°C in stationary phase cells. The presence of tetracycline in the growth medium at 28°C induced *emhABC *by ~10-fold. Induction levels obtained for all these conditions were significantly different (P < 0.005) from the calibrator. In each case, except for logarithmic growth, the three *emhABC *genes were expressed at equivalent levels, but during log phase their expression followed the trend *emhA > B > C*.

Expression of *emhABC *genes did not increase in stationary phase cells incubated at 28°C in the presence of chloramphenicol, naphthalene or phenanthrene although chloramphenicol and phenanthrene are known substrates of EmhABC efflux pump. This is consistent with the hypothesis that PAHs and antibiotics are not primary substrates of resistance-nodulation-division (RND) efflux pumps [6,7]. The observation by Hearn et al. [18] that *emhABC *genes are not induced by PAHs was confirmed by the current study. Conversely, antibiotics such as tetracycline and chloramphenicol that inhibit ribosomal function were shown to induce the expression of *mexY*, which encodes the MexY efflux pump in *P. aeruginosa *PAO1, but their effect on expression was concentration-dependent [8]. Induction *of emhABC *by tetracycline but not chloramphenicol (Figure 3) may likewise depend on concentration. Because single sub-lethal concentrations of antibiotics were tested in this study we cannot make any conclusions about the effect of chloramphenicol on *emhABC *expression. Alternatively tetracycline may be a better substrate of the EmhABC efflux pump able to induce its expression compared to chloramphenicol and phenanthrene. Dimethylformamide, the water-miscible solvent used to add the PAHs, did not affect expression of *emhABC *genes in parallel control incubations (results not shown).

### Incubation temperature affects cLP6a membrane integrity

**Figure 4:**
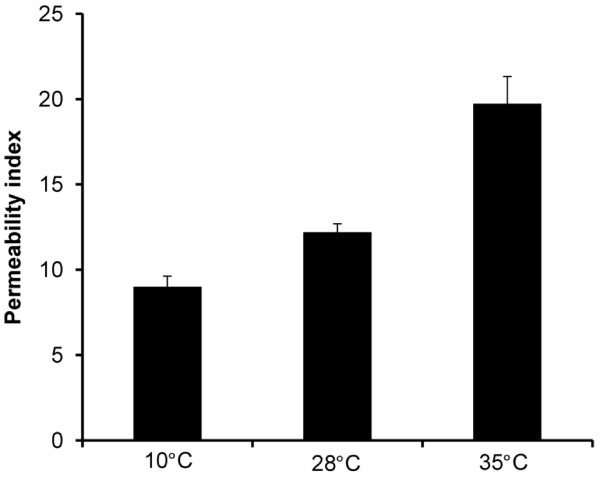
**The permeability index *of P. fluorescens *cLP6a**. The permeability index of *P. fluorescens *cLP6a cells grown to stationary phase at 10°C, 28°C or 35°C. See text for definition of permeability index. Each bar represents the mean of three culture sub-samples.

Because the activity of EmhABC was low but the expression of *emhABC *was high in cLP6a cells grown at 35°C compared to other incubation temperatures, we hypothesized that membrane integrity and (or) changes in membrane FA components might be responsible for these observations. To test the hypothesis, cell membrane integrity was determined using fluorescent dyes to determine the effect of incubation temperature on membrane permeability. Propidium iodide (PI) is a fluorescent reporter molecule that cannot cross intact cell membranes [23]. Therefore, cell fluorescence in the presence of PI only occurs if membrane integrity is compromised, allowing PI to penetrate and interact with intracellular DNA. Cetyltrimethylammonium bromide (CTAB) is a cationic surfactant that can permeabilize bacterial cell membranes and thus increase PI penetration. The fluorescence value of cells exposed to PI with CTAB treatment or without CTAB treatment represents, respectively, the total number of cells (with artificially induced membrane permeability) and the number of cells naturally exhibiting compromised membrane integrity [23]. A permeability index can be calculated as the percentage of the net fluorescence value of PI-treated cells in the absence of CTAB relative to that in its presence. In Figure 4 the permeability index of cLP6a cells grown to stationary phase increased with higher incubation temperature: cells grown at 10°C, 28°C or 35°C had permeability indices of approx. 9%, 12% and 20% respectively. This indicates that, as anticipated, cLP6a cells exhibit increasingly compromised membrane integrity when grown at 35°C, just below the maximum permissive growth temperature.

### Membrane FA content is modified in response to incubation temperature

Temperature is well known to cause modification of membrane FA content [11,12]. Therefore, the membrane FA profiles of strain cLP6a grown to stationary phase at 10°C, 28°C or 35°C, in the presence of PAHs or antibiotics were quantified to determine the effect of temperature on cell membrane FA composition (Table 3). Strain cLP6a grown at 28°C in the absence of PAHs and antibiotics was used as a reference. Generally, incubation temperature caused greater changes in the proportions of saturated-, unsaturated- and cyclopropane-FA than the other conditions tested. Compared to 28°C, cells grown at 10°C responded by decreasing the total saturated membrane FA by half to ~20%, decreasing cyclopropane-FA from 43% to 7% and concomitantly increasing total unsaturated FA from 14% to 72%, primarily represented by the *cis*-isomers of 16:1Δ9 and 18:1Δ9. Cells grown at 35°C responded with slight increases in total saturated and cyclopropane-FA and a 4-fold decrease in total unsaturated FA. In the presence of tetracycline, cLP6a cells responded with a ~2-fold increase in unsaturated membrane FA and a ~25% decrease in total cyclopropane-FA but unchanged total saturated membrane FA. There were no major changes in the proportions of different membrane FA in cells incubated with chloramphenicol, naphthalene or phenanthrene. Consistent with observations *of emhABC *gene induction, tetracycline but not chloramphenicol induced major changes in membrane FA content (although both antibiotics are substrates of EmhABC), possibly due to the sub-inhibitory concentration of chloramphenicol used in the assay or because tetracycline is a better substrate of EmhABC efflux pump. In contrast, the PAHs naphthalene and phenanthrene did not induce major FA changes likely because cLP6a is adapted to growth on PAHs, having been isolated from a hydrocarbon-contaminated soil [16].

**Table 3:** FA composition of *P. fluorescens *strain cLP6a under different growth condition

	FAs as% of total FA detec ted *													
				
Growth conditions	14:0	15:0	16:0	16:1Δ9c	16:1Δ9t	17:0	cy17	18:0	18:1Δ9c	18:1Δ9t	Cy19	Total Saturated FAs	Total Unsaturated FAs	Total Cyclo-FAs
10°C	0.2	0.2	19.9	34.0	7.0	0.3	6.6	0.3	30.5	0.7	0.4	**20.9**	**72.2**	**7.0**
28°C	1.0	0.2	40.4	4.6	1.6	0.3	40.0	1.2	7.6	ND^**†**^	3.1	**43.1**	**13.8**	**43.1**
35°C	0.6	0.2	44.6	1.3	0.1	0.3	44.1	1.9	2.1	0.1	4.9	**47.6**	**3.6**	**49.0**
28°C with naphthalene	0.6	0.1	40.8	5.5	3.2	0.2	36.5	1.2	9.3	0.3	2.3	**42.9**	**18.3**	**38.8**
28°C with phenanthrene	0.7	0.2	40.1	4.7	1.9	0.3	39.7	1.2	7.9	ND	3.3	**42.5**	**14.5**	**43.0**
28°C with tetracycline	1.0	0.2	40.3	14.5	ND	0.3	32.5	1.0	8.6	ND	1.6	**42.8**	**23.1**	**34.1**
28°C with chloramphenicol	1.1	0.2	41.0	6.6	ND	0.4	40.1	1.3	6.2	ND	3.1	**44.0**	**12.8**	**43.2**

Strain cLP6a cultures were grown to stationary phase at 10°C, 28°C or 35°C, or grown at 28° in the presence of PAHs (naphthalene or phenanthrene, at 5 mmol l^-1^) or antibiotics (tetracyclin or chloramphenicol, at 1/4 MIC). FA contents are expressed as the mean weight % of total FA detected in two measurements; deviation from the mean was typically <1% and at most 3% of the measured values.

*, FA nomenclature: number of carbons; saturation (:0); mono-unsaturation (:1); position of double bond calculated from the carboxyl end (Δ9); *cis- *(c) or *trans- *(t) isomer; cyclopropyl ring (cy)

†, not detected

### Free FA are substrates of EmhABC

**Figure 5:**
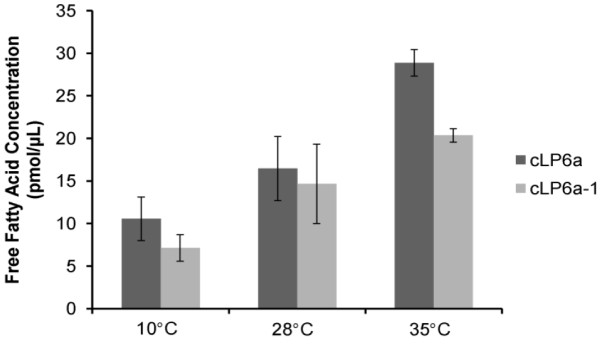
**Free FA in cell free medium of *P. fluorescens *strains cLP6a and cLP6a-1 cultures**. Free FA concentration in filtered medium from cLP6a and cLP6a-1 cultures grown to stationary phase at 10°C, 28°C or 35°C. Each bar represents the mean of two independent experiments, and error bars, where visible, indicate the average deviation.

We investigated the possibility that free FA released from membranes damaged by stress or undergoing rapid phospholipid replacement are substrates of the EmhABC efflux pump. The concentration of free FA was determined in the cell-free medium of strains cLP6a and cLP6a-1 grown at 10°C, 28°C or 35°C to stationary phase. The concentrations of free FA in the cell-free medium of cLP6a and cLP6a-1 cultures incubated at 10°C or 28°C (Figure 5) were not significantly different (*P *< 0.4 or *P *< 0.8 respectively). However, there was a significant difference (*P *< 0.04) in the concentration of free FA in the medium of cLP6a and cLP6a-1 cultures incubated at 35°C. Higher concentrations of free FA were observed in the medium of cLP6a cultures grown at 35°C in the presence of a functional EmhABC pump compared to cultures of cLP6a -1 lacking EmhABC, consistent with the involvement of EmhABC in the transport of FA originating from membranes under stress or rapid turnover.

## Discussion

Efflux pumps of the resistance-nodulation-division (RND) superfamily are common in Gram negative bacteria [7,28] and are well studied for their role in antibiotic resistance and solvent tolerance in many *Pseudomonas *species [29,30]. However, these may not be the native or dominant physiological functions of RND pumps in bacteria. Piddock [6] and Poole [7], among others, have suggested that RND pumps fulfill other crucial roles, including management of diverse physico-chemical and biochemical stresses, quorum sensing and virulence. One of the stress-responsive roles proposed for RND efflux pumps such as MexCD-OprJ in *Pseudomonas aeruginosa *[4,7,31] is the export of membrane constituents released by FA replacement due to natural turnover of membrane components during cell growth or resulting from membrane damage. Our results are consistent with that proposal: EmhABC appears to play a role in efflux of replaced membrane FA in response to temperature-induced membrane perturbation, in addition to its demonstrated function of transporting hydrophobic antibiotics, dyes and PAHs [18]. Reciprocally, because RND efflux pumps are membrane-associated protein complexes, EmhABC activity may in turn be influenced by modulation of FA content in response to membrane stressors like temperature and hydrophobic compounds [11] that partition into lipid bilayers. As expected, our results indicate that temperature affects cell growth, FA content (measured as fatty acid methyl esters) and membrane integrity (measured as permeability index). In addition, we have now shown that temperature affects expression and activity of the EmhABC RND efflux pump (measured by using RT-qPCR, phenanthrene efflux and antibiotic MIC assays).

The FA content of cLP6a followed the expected trends at 10°C and at 35°C, shifting towards unsaturation and saturation respectively [11,32]. The FA content of the membrane affected the partitioning of phenanthrene into the membrane, since cLP6a-1 cells grown at 35°C contained lower fractions of phenanthrene in the absence of active efflux compared to those grown at 28°C. This observation is consistent with the rationale that saturated FA pack closely, hindering partitioning of hydrophobic molecules like PAHs into the lipid bilayer [11] whereas angular *cis*-unsaturated FA pack more loosely, facilitating partitioning. The observed changes in FA with temperature are also consistent with results from the membrane integrity assay in which the permeability index increased with temperature.

Growth temperature also affected EmhABC activity in cLP6a, possibly indirectly through membrane perturbation including the modulation of FA. cLP6a cells having high unsaturated FA content (i.e., 72% in cells grown at 10°C) and greater membrane integrity had higher efflux activity than cells with lower proportions of unsaturated FA (i.e., 14% at 28°C or 4% at 35°C) and increased permeability. This observation suggests that increased unsaturated FA content may allow efficient or stable association of the three protein components of RND efflux pumps, which spans two membranes and the periplasm.

The enhanced phenanthrene efflux observed in cLP6a at 10°C is consistent with the additive effect of EmhABC with a postulated alternate efflux pump that is active at 10°C. The presence of an alternate pump in *P. fluorescens *is not unexpected, as multiple efflux pumps have been identified in other *Pseudomonas *species [2,7] and additional efflux pumps were invoked by Hearn et al. [18] to explain anthracene and fluoranthene efflux in *P. fluorescens *strain cLP6a.

The induction *of emhABC *genes was observed in cLP6a cells exhibiting major changes in membrane FA composition due to sub-optimal growth conditions, namely at 10°C, 35°C and in the presence of tetracycline. Expression was also increased in logarithmic phase cells, which undergo rapid synthesis and turnover of FA, and in death phase cells that experience membrane deterioration. The relationship between induction *of emhABC *genes and membrane FA modulation indicates that the EmhABC efflux pump may be involved in the extrusion of replaced membrane FA as a result of membrane turnover. This possibility is further supported by the higher concentration of free FA in the medium of cLP6a cultures grown at 35°C concomitant with high membrane permeability and over-expression of *emhABC *genes. Comparable results were obtained recently by Stickland et al. [31] who reported that over-expression of *mexCD-oprJ *efflux genes in *P. aeruginosa *led to up-regulation of FA secretion and fitness impairment. Over-expression of *emhABC *genes in cLP6a cells grown at 35°C may be explained either as compensation for reduced activity of EmhABC (caused by the modulation of the FA content) or may be due to increased membrane permeability and membrane FA turnover. According to Denich et al. [11], damage to the membrane is still possible even with modulation of membrane FA quantity or composition to maintain fluidity and integrity. Our conclusion is supported by the observation of similarly high levels of *emhABC *over-expression in log phase cells. Such cells may have compromised cell membranes due to rapid phospholipid synthesis and turnover since membrane integrity is temporarily affected by physical cell wall reconstruction at the sites of cell division during the log phase of growth [33,34]. It is unclear why there was differential expression of the three *emhABC *genes in log phase cells (*emhA *>*B *>*C*), although stability of the transcripts may differ as a result of rapid cell growth. The effect on membrane integrity was confirmed by the higher permeability index at 35°C. Similarly, the reduced cell yields and growth rates at 35°C compared to 10°C or 28°C, along with altered FA content, are consistent with compromised cell membranes at the higher temperature. The negative effects of the compromised membrane on growth are muted by the presence and activity of EmhABC, allowing cLP6a cells to out-grow cLP6a-1 at supra-optimal temperature.

The discovery that EmhABC activity influences growth of *P. fluorescens *cLP6a (and by extension wild type LP6a) at supra-optimal temperature suggests a role for efflux in temperature adaptation in the environment, and may apply to other Gram-negative species. For example, *P. aeruginosa *and *Salmonella *strains lacking RND efflux pumps are unable to colonize and infect their hosts [1,35], which may in part result from an inability to adapt to host temperatures higher than the external environment. Temperature also may affect efflux-mediated antibiotic resistance although the effect on MIC was not pronounced in *P. fluorescens *cLP6a. It will also be interesting to examine whether temperature-sensitive efflux of antibiotics is a general phenomenon in other Gram-negative bacteria. Because bacterial cells are commonly exposed to temperature changes in the environment, we propose that RND efflux pumps in Gram-negative bacteria may play a major role in management of temperature-induced membrane damage.

Our study focussed on modifications to the FA portion of membrane lipids since phospholipid head group modification is typically less dynamic and critical in bacteria (reviewed by Denich et al. [11]), but it is possible that head group composition also changed in response to temperature, PAHs and/or antibiotics. Other indirect effects such as decreased proton motive force resulting from damaged membranes could also be factors. Such possibilities are incentives for clarifying the natural physiological roles of RND efflux pumps in Gram-negative bacteria in anticipation of devising new methods for combating antibiotic resistance or improving hydrocarbon transformation for bioremediation or biocatalytic processing of hydrophobic substrates.

## Conclusions

The alternative and likely the primary physiological role of EmhABC in *P. fluorescens *cLP6a is the efflux of membrane FA replaced as a result of adaptation to membrane stress caused by physico-chemical stressors. Efflux of unnatural substrates such as hydrophobic antibiotics, PAHs or dyes may be a consequence of membrane stress.

## Authors' contributions

AAA designed and performed all experiments, acquired, analysed and interpreted data and drafted the manuscript. JMF conceived of the study, participated in its design and coordination, helped draft and critically revise the manuscript. All authors read and approved the final manuscript.
